# P-695. Heath-related quality of life in children with respiratory infection

**DOI:** 10.1093/ofid/ofaf695.908

**Published:** 2026-01-11

**Authors:** Taito Kitano, Sayaka Yoshida

**Affiliations:** Nara prefecture general medical centre, Shinjuku, Tokyo, Japan; Nara Prefecture General Medical center, Nara, Nara, Japan

## Abstract

**Background:**

Respiratory infection brings enormous morbidity and mortality among children. A quantitative evaluation of disease burden of respiratory infection using health-related quality of life (HRQL) helps accurate health technology assessment and cost-effectiveness analysis regarding preventive and therapeutic strategies associated with respiratory infection. The objective of this study was to evaluate the impact of respiratory infection on pediatric HRQL by different respiratory pathogens.

**Methods:**

HRQL of children younger than 16 years with respiratory infection was measured using proxy versions of EuroQol 5-dimensions youth (EQ-5D-Y) for aged 4–15 years, EuroQol Toddler and Infant Populations (EQ-TIPS) for aged 0–3 years, and Pediatric Quality of Life Inventory (PedsQL) for aged 0–15 years. The impacts of respiratory infection on pediatric HRQL were presented as level sum score for EQ-TIPS (higher score is more problem), utility value for EQ-5D-Y (higher score = better health status), and total score for PedsQL (higher score = better health status). The results were analyzed based on detected respiratory viruses.

**Results:**

In total, parents or caregivers of 738 eligible children were offered to participate in the study, of which answers were obtained from 540 participants (266 of children aged 0–3 years and 274 from those aged 4–15 years). In children aged 0–3 years, rhinovirus (51.1%) was the most frequently detected, followed by respiratory syncytial virus (33.5%). In those aged 4–15 years mycoplasma (39.4%) was the most common respiratory pathogen. Overall, the median EQ-5D-Y score in those aged 4–15 years with respiratory infection was lowest in post-symptomatic day 2 (0.753) and day 3 (0.753), compared with that in pre-symptomatic (0.953), which gradually improved to the presymptomatic level at day 9 (0.953). By respiratory pathogen, the largest reduction in median PedsQL score in the first symptomatic week, compared with the pre-symptomatic week was observed in RSV (-32.1) among children aged 0–3 years and mycoplasma (-36.4) among children aged 4–15 years.

**Conclusion:**

Among multiple respiratory pathogens, RSV in young children and mycoplasma in older children had a large impact on pediatric quality of life score while symptomatic.

**Disclosures:**

All Authors: No reported disclosures

